# Supplementary material for: Learning curve and surgical outcome of robotic assisted colorectal surgery with ERAS program
Source: Sci Rep. 2022 Nov 29;12:20566. doi: 10.1038/s41598-022-24665-w (PMC9709162; doi:10.1038/s41598-022-24665-w)
Supplement: Supplementary file 2 — Supplementary Information 2. [file 41598_2022_24665_MOESM2_ESM.zip › RAL Raw data-Ñ[▒K/128/HS_ñw╝╨░O▒Kñσ.pdf]

正式摘要(電子簽章：000000230880901)	☐【讀取】憑證	
W52 / W52 ☐英文標題(注意:無英文姓名-請補病人基本檔!)		

臺中榮民總醫院

出院病歷摘要

轉入號： 轉出號：

[redacted]

(8)流水編號	
(9)入院日期	110/11/19 (2021) 直腸外科 W52 - 161
(10)轉科日期	
(11)出院日期	110/11/25 (2021) 住院天數計 6 日

診斷

- 入院：1. Rectal cancer, 7cm FAV, cTxNxM0,
  - s/p short course Radiotherapy(11/15-11/19)
- 出院：1. Rectal cancer, 7cm FAV, cTxNxM0,
  - s/p short course Radiotherapy(11/15-11/19)
  - s/p Robotic LAR + loop-ileostomy + Left D-J insertion on 2021/11/20

主訴

- Bloody stool for months

病史

- This is a 54 years old male patient with history of

  1. Hemorrhoid s/p OP in 2021/09

  According to the patient, he had diarrhea for 1 year and bloody stool had been noticed. He visit LMD and treated as Hemorrhoid, received operation in 2021/09. But the symptoms persist after op, so the LGI scopy was arranged at LMD, and showed rectal tumor, 7cm FAV, biopsy reported adenocarcinoma. Then, he visited our CRS OPD for second opinion. After discussion, he received short course radiotherapy first, and this time, he was admitted to our ward for scheduled operation.

體檢發現

- PS 70%
  1101117 No side effect
  - General appearance :
    - easy looking , conscious clear , without cardiopulmonary distress
    - Vital signs自動帶入date 體溫(BT) 脈搏(P) 呼吸(R) 血壓(BP) 身高(BH) 體重(BW) BMI
      20211119 36.4℃ 78次/分 20次/分 103/59mmHg 164.3cm 65.5kg 24.3
    - Vital signs: B.P. 103/59 mmHg , P.R. 78 /min , R.R. 20 /min , B.T. 36.4 ℃
  - HEENT :
    - Eyes: no anemic conjunctiva, no icteric sclera, normal EOM
    - ENT: no otorrhea, no rhinorrhea, no erythematous mucosa, no injected tonsil, no deformity
  - Neck: supple, freely movable, trachea midline,no deformity, no engorged jugular vein , no palpable thyroid or lymph node enlargement
  - Chest:
    - Inspection: Grossly normal, symmetric and fully expansion, no accessory muscle used, no operation scar, no spider angiomata, no gynecomastia
    - Palpation: no palpable mass or nodule, no subcutaneous emphysema, no tenderness
    - Ausculation: clear breathing sound
  - Heart:
    - Palpation: no thrill, no heave
    - Percussion: normal heart size
    - Ausculation: regular heart beat, normal S1, S2, no gallop, no murmur

- Extremities: No deformity, full ranged of motion without limitation, no clubbing finger or nail cyanosis, no pitting edema, no ulcer or pigmentation, no obvious skin lesion, no varicose vein, no bone, joint or muscle tenderness

CRS CONDITION

- Karnofsky performance status: (100) Normal, no complaints, no evidence of disease
- PE of Abdomen:
- CEA: 14.88 ng/ml
- Imaging Studies:
- Colonoscope report: .
- Figures:
  1. colon & rectum

手術日期及方法

- 手術日期及方法20211120 Ureter, Double J insertion
  20211120 1. Robotic (XI) assisted TME+ loop ileostomy+ L't D-J insertion
  2. Intraoperative colonoscopy : (-).
  3. High ligation (+) : At IMA root (Via robotic clips). IMV was ligated via endoclip.
  4. Take down from the splenic flexure : (-)
  5. Robotic EndoGIA (45-4.3) X2 + EEA (29mm) + OpenGIA (80-3.8) X1 were applied to complete the side-to-end anastomosis
  6. Proximal cut end: > 10 cm, distal cut end: 3 cm
  7. No drainage tube was inserted
  8. ADHESION BARRIER FILM was placed
  9. Loop ileostomy (About 15~20cm from IC valve) was done over RLQ of abdomen
  10.frozen pathology was sent(peritoneum)
  11.D-J insertion by GU doctor

住院治療經過

- After admission, he received Robotic LAR + loop-ileostomy + Left D-J insertion on 2021/11/20. He start try bland II diet since 11/22 and shift abx to Subacillin. He start try soft diet since 11/23 and tolerate. Foley, CVC and Rod were removed on 11/24. Under relative stable condition, he was discharged on 11/25, OPD f/u.

合併症

一般檢查

- 累積報告 :

DATE	TIME	ALB	TP	BIL.T	BIL.D	ALKP	AST	ALT	LDH	AMY	LIPASE	NH3	Ferritin	UA
1101120	0616		5.3											4.2
1101120	1658	3.0		0.4				12						
1101122	1053			0.68			16							

DATE	TIME	WBC	RBC	HGB	HCT	MCV	PLT	BLST	NEUT	BAND	SEG	LYM	MON	EOS	BAS
1101119	1628	6030	3.91	8.0	27.1	69.3	417		81.2			11.8	5.8	1.0	0.2
1101120	0611			9.2	30.5										
1101120	1658	11420		9.1	30.8		369		94.1			1.7	3.7	0.1	0.4
1101122	1120	16730		10.1	33.9		380		93.3			2.2	4.1	0.0	0.4

DATE	TIME	NA	K	CL	BUN	CREAT	eGFR	CA	Mg	P	CRP	ESR	PCT	D-Dimer	LACTATE
1101120	0616										0.664				
1101120	1658	141	3.7	107	12	0.83	102.62	8.1							
1101122	1046														12.7
1101122	1053	136			10	1.02	80.89								

DATE	TIME	GLUCOSE	HbA1C	CHOL	TG	HDL	LDL	KETONE	CK	CKMB	TRO-T	PT-P	APTT-P	NT-pro BNP
1101120	0615	218												
1101120	0616			123	72	38	76							

特殊檢查

病理報告

其它

出院時情況

- 3.改門診治療

出院指示

藥名	劑量	劑量單位	用法	頻次	數量	數量單位	現況	天數	單位
Unasyn TAB 375mg	1	TAB	PO	Q12H	6	TAB	TH	3	天
(口)Takepron OD tab 30mg	1	TAB	PO	QDAC	7	TAB	TH	7	天
Ultracet tab	1	TAB	PO	Q6H	28	TAB	TH	7	天
Celebrex cap 200mg	1	CAP	PO	BID	14	CAP	TH	7	天
TramTOR cap 50mg	1	CAP	PO	Q6HPRN	28	CAP	TH	7	天

出院計劃

- 門診日期
  - date on 2021/12/01

主治醫師 2062F 蔣鋒帆 / 住院醫師 2339C 蔡濬樟

正式摘要(電子簽章：000000230880901)

W52 / W52

doctorRank:B
